# Prospective Pilot Study Comparing the Need for Adaptive Radiotherapy in Unresected Bulky Disease and in Postoperative Patients With Head and Neck Cancer

**DOI:** 10.1177/1533034617717624

**Published:** 2017-07-03

**Authors:** Omar Mahmoud, Isildinha M. Reis, Michael M. Samuels, Nagy Elsayyad, Elizabeth Bossart, Joseph Both, Ehsan ELGhoneimy, Magda Moustafa, Mohamed AbdAllah, Cristiane Takita

**Affiliations:** 1 Kasr El-Aini Center of Radiation Oncology and Nuclear Medicine (NEMROCK), Cairo, Egypt; 2 Department of Radiation Oncology, Rutgers, The State University of New Jersey, New Brunswick, NJ, USA; 3 Division of Biostatistics, University of Miami Miller School of Medicine, Miami, FL, USA; 4 Department of Radiation Oncology, University of Miami Miller School of Medicine, Miami, FL, USA

**Keywords:** adaptive radiotherapy, dosimetric, head and neck cancer, postoperative chemoradiotherapy, definitive chemoradiotherapy

## Abstract

**Background:**

Adaptive radiotherapy is being used in few institutions in patients with head and neck cancer having bulky disease using periodic computed tomography imaging accounting for volumetric changes in tumor volume and/or weight loss. Limited data are available on ART in the postoperative setting. We aim to identify parameters that would predict the need for ART in patients with head and neck cancer and whether ART should be applied in postoperative setting.

**Materials and Methods:**

Twenty patients with stage III–IV head and neck cancer were prospectively accrued. A computed tomography simulation was done prior to treatment and repeated at weeks 3 and 6 of concurrent intensity-modulated radiotherapy and chemotherapy. The final plan was coregistered with the subsequent computed tomography images, and dosimetric/volumetric changes at weeks 1 (baseline), 3, and 6 were quantified in high-risk clinical target volumes, low-risk clinical target volumes , right parotid , left parotid , and spinal cord . An event to trigger ART was defined as spinal cord maximum dose >45 Gy, parotid mean dose >26 Gy, and clinical target volume coverage <95%.

**Results:**

Comparing the 2 groups, the proportion of patients with at least 1 event triggering ART was higher in bulky disease than in postoperative group: 72.7% versus 18.2% (*P* = .03) overall; 54.6% versus 1.8% (*P* = .064) at week 3; and 63.6% versus 18.2% (*P* = .081) at week 6. In the bulky disease group, 8 of 11 patients had events at week 3 and/or 6 as follows: overdose in spinal cord (n = 2), right parotid (n = 3), left parotid (n = 5), coverage < 95% seen in low-risk clinical target volumes (n = 3), and high-risk clinical target volumes (n = 5). In the postoperative group, 2 of 11 patients had events: spinal cord (n = 1) and low-risk clinical target volume (n = 1).

**Conclusion:**

Our study confirmed the need for ART in patients with head and neck cancer having bulky disease due to target under dosing and/or spinal cord/parotids overdosing in weeks 3 and 6. In contrast, the benefit of ART in postoperative patients is less clear.

## Introduction

The sharp dose gradient of intensity-modulated radiotherapy (IMRT) allows greater normal tissue sparing, thus improving quality of life in patients with head and neck cancer (HNC).^1^ In the definitive and adjuvant setting, patients receiving IMRT with chemotherapy may experience weight loss, tumor shrinkage, and/or parotid shrinkage, which may result in significant volumetric^2,3^ and dosimetric changes^4,5^ compromising IMRT plan quality. The IMRT plan is generated on baseline CT images, which would not account for the changes occurring throughout the treatment. The adaptive radiotherapy (ART) concept entails reimaging the patients during the radiotherapy course and generating a new adaptive plan to accommodate these new changes.^6,7^ The ART is still evolving and is not considered routine practice as questions remain to be answered regarding proper patient selection, magnitude of benefit, rescan timing and frequency, increase in workload, and optimization/automation of adaptation tools.^8^

The majority of studies tried to assess the value of ART in patients with HNC treated on definitive basis.^9^ To date, one study specifically addressed the selective use of mid-treatment ART in postoperative (PO) patients with HNC and suggested minimal benefit.^10^ Due to the paucity of studies addressing the role of ART in this patient population, we aimed, through this prospective study, to track the volumetric/dosimetric changes comparing PO patients with HNC and patients with bulky disease (B) having HNC. In addition, we aimed to detect the impact of weight loss and its potential threshold value to initiate ART.

## Materials and Methods

### Patient Selection

A total of 22 patients were enrolled in this prospective observational study from 2010 to 2012. The study was approved by the institutional review board and all patients signed informed consent. Patients with biopsy-proven American Joint Committee on Cancer 2010 stages III or IV squamous cell carcinoma of the nonsalivary and nonparanasal sinus HNC who were to receive IMRT were included. All of the 11 PO patients received chemoradiotherapy (ChRT). Definitive ChRT was delivered to the other 11 patients with B defined as having gross tumor measuring more than 4 cm.

### Initial Image Acquisition and Contouring

All patients, B and PO, were immobilized with a customized thermoplastic head and shoulder mask (Orfit; Orfit Industries, Jericho, New York). Contrast-enhanced CT scans were obtained on a spiral CT scanner using 3-mm slice intervals. Eclipse treatment planning system (Varian Oncology Systems, Palo Alto, California) was employed for target and normal structure delineation and to generate IMRT arc plans. The treating physician manually contoured the target volumes and normal structures for each patient on the baseline planning CT scan using the RTOG Head and Neck Atlas guidelines. Diagnostic head and neck MRI and/or PET images were fused with the baseline CT planning, when available, in patients with B . Clinical or radiographic tumor and/or enlarged lymph nodes visible on CT, MRI, or PET were contoured as gross tumor volume (GTV). As the protocol did not mandate additional PET or MRI reimaging at weeks 3 and 6, GTV recontouring was not performed. The high-risk clinical target volume (CTV-HR) included the regions and/or subjacent lymph node chains within 2 to 3 cm of gross disease. The low-risk clinical target volume (CTV-LR) included the uninvolved non-subjacent neck node chains still at a risk of containing micrometastasis determined by knowledge of the natural history of the disease. A 3 mm expansion of each CTV was applied to create the respective planning target volume (PTV). The PTV contours were modified to eliminate portion extending beyond skin surface. The IMRT dose normalization value was selected to ensure 100% of prescription dose coverage to >95% of each PTV. For the PO group, a dose of 60 to 66 Gy was prescribed to high-risk planning target volume (PTV-HR) and 54 Gy to low-risk planning target volume (PTV-LR) in 30 to 33 fractions. For the B group, 59.4 Gy was prescribed to PTV-HR while 54.45 Gy was prescribed to PTV-LR in 33 fractions. Simultaneous integrated boost was used for all IMRT single-arc plans. In the B and PO groups, CTV-HR excluded the volume getting higher dose and no overlap was allowed with GTV treated to 69.96 Gy in 33 fractions or areas receiving 66 Gy in the PO group (in case of extracapsular extension or positive surgical margins). All initial IMRT plans (before treatment initiation) met dose constraints; 95% of PTV covered with the prescription dose, while keeping the mean dose to each parotid below 26 Gy and the maximum spinal cord (SC) dose below 45 Gy.

### Recording of Dosimetric and Volumetric Changes on Repeat Imaging

Two additional planning CT scans in the treatment position were obtained at treatment week 3, during fraction 15 (±2), and at week 6, fraction 27 (±2). Automatic rigid registration followed by fine manual adjustment of the initial CT fusion to the subsequent CT was achieved using bony landmarks and the external patient contour. Target volumes and normal structures were copied from the original CT to be modified on the newer image sets based on anatomical changes. These modified contours executed by the treating physician performed better than available soft registration software, adjusting for volumetric changes (not only to air tissue interface) and, accordingly, adopted as the method of choice for the tracking of volumetric/dosimetric changes. The original IMRT plan was copied on subsequent CT image sets by applying the beam configurations of the initial IMRT plan to the anatomy of subsequent CT scans. Based on the generated data, some patients continued to the treatment with an adapted new plan—based on the discretion of the treating physician—if target volumes coverage dose falling below 95%, mean dose to parotid gland exceeding 26 Gy, and/ or SC maximum dose more than 45 Gy. These parameters—originating from national practice guidelines^11^—were recorded as dose deviations triggering ART. Regardless of the adapted plan applied in some patients, the nonadapted dose distribution of the original IMRT plan was used as a reference for comparison with subsequent image sets in all the accrued patients.

Volumetric changes occurring at weeks 3 and 6 were recorded for the following structures: CTV-HR, CTV-LR, right parotid (RP), and left parotid (LP). The resultant dosimetric changes were collected as follows: mean dose to CTV-HR, CTV-LR, RP, LP, and SC maximum dose. On repeat imaging, CTV-HR and CTV-LR dose coverage falling below 95%, mean dose to parotid gland exceeding 26 Gy, and/ or SC maximum dose more than 45 Gy were recorded as dose deviations triggering ART. In the B and PO groups, these deviations were counted both in weeks 3 and 6. The patients’ weight was also recorded at the same time to evaluate for volumetric changes as a potential clinical indicator signaling ART initiation.

### Statistical Analysis

Volumetric and dosimetric data were obtained at baseline, week 3, and week 6. Raw data and percentage change relative to baseline (= 100 × change/baseline) were summarized as means, standard deviation, and standard error. Absolute and percentage change variables at week 3 and week 6 relative to baseline, and the corresponding differences, were analyzed by a 2-way repeated-measures analysis of variance model using SAS MIXED procedure. The repeated measures model included group (B versus PO) as a fixed factor, week (3 and 6) as a repeated-measures factor, and their interaction. Tests for comparison between groups at weeks 3 and 6, as well as within-group comparisons of absolute or percentage change for week 3 versus week 6, were performed using contrasts. To evaluate the changes relative to baseline, we tested whether each absolute or percentage change mean was significantly different from zero. To evaluate the dose deviations that trigging the ART event, the Fisher exact test was used for group comparison with respect to categorical outcomes at specific week. Pearson correlation analysis method was conducted to estimate linear correlation between clinical and dosimetric factors such as weight loss and parotids/target volume shrinkage. *P* value ≤.05 was considered significant. The magnitude of changes was compared in both groups (B vs PO). Another set comparison was applied within each group comparing the magnitude of changes occurring at week 3 versus week 6. Analyses were conducted in SAS version 9.3 (SAS Institute, Cary, North Carolina).

## Results

### Patient Characteristics

After accruing 36 patients, 22 consecutive patients with complete data with locally advanced HNC treated with ChRT were accrued to this prospective observational study between February 2011 and December 2012. The patient and tumor characteristics are shown in Table 1.

**Table 1.** Patient and Tumor Characteristics.

Variable	N (%) = 22 (100.0)
Age, years	
40 to 59	11 (50.0)
60 to 69	6 (27.3)
≥70	5 (22.7)
Gender	
Female	16 (72.7)
Male	6 (27.3)
T stage	
T1	5 (22.7)
T2	10 (45.5)
T3	3 (13.6)
T4a	3 (13.6)
T4b	1 (4.5)
N stage	
N0	2 (9.1)
N1	3 (13.6)
N2a	3 (13.6)
N2b	13 (59.1)
N3	1 (4.5)
AJCC group stage	
Stage III	5 (22.7)
Stage IVA	14 (63.6)
Stage IVB	3 (13.6)
Primary site	
Oropharynx base of tongue	6 (27.3)
Oropharynx tonsil	5 (22.7)
Oral cavity tongue	3 (13.6)
Oral cavity (other Sites)	4 (18.2)
Larynx supraglottic	2 (9.1)
Hypopharynx	1 (4.5)
Nasopharynx	1 (4.5)
Study group	
Postoperative (PO)	11 (50.0)
Bulky (B)	11 (50.0)

Abbreviations: AJCC, American Joint Committee on Cancer.

### Volumetric and Dosimetric Changes During Radiotherapy

Compared to baseline, there was significant weight loss in both groups at weeks 3 and 6 (Table 2). There was a trend toward a higher weight loss in group B compared to PO at week 6 (percentage change relative to baseline 8.6% vs 4.7%, *P* = .053). The volumetric changes in both B and PO patients at week 3 and 6 are shown in Table 3. The mean percentage shrinkage in CTV-HR volume relative to baseline was statistically significant in group B at weeks 3 and 6, respectively (7.2%, *P* = .002 and 12.8%, *P* = .0002), and their difference was also significant (5.7%, *P* = .043). In contrast, group PO did not show significant CTV-HR volume shrinkage from week 3 to week 6 but only relative to baseline (7.8%, *P* < .0001, and 10.9%, *P* = .0009, difference 3% NS). The difference in mean volumetric reduction in CTV-LR at week 6 versus 3 approached significance in group B (4.1% vs 7.9%, difference 3.8%, *P* = .058), and there was no difference in the PO group. The parotid volumes were significantly reduced at both weeks 3 and 6 as compared to baseline in both groups (mean reductions range from 10% to 30.9%, all at *P* <.0001), with a slight higher percentage of reduction in the B group. However, group differences were only statistically significant for LP volume. The RP volume demonstrated reductions in groups B versus PO of 19% versus 16.6% (difference 2.3% NS) at week 3 and 30.9% versus 25.3% (difference 5.6% NS) at week 6. The LP volume shrinkage was significantly higher in the B group when compared to the PO group in both weeks: mean volume shrinkage 18.2% versus 10% (8.2%, *P* = .008) at week 3 and 30.1% versus 23.1% (7%, *P* = .04) at week 6.The volumetric changes in OAR and CTVs had minor impact on dosimetric changes seen in groups B and PO, as summarized in Table 4. Despite minimal increase in the mean target dose observed in CTV-HR and CTV-LR at week 3 and week 6 in both groups, the target coverage dropped below 95% in 6 patients with B compared to only 1 PO patient as seen in Table 5. The mean dose increase in both CTV-HR and CTV-LR was 1.5 Gy or lower. The maximum SC dose increase at week 3 was marginally higher in group B compared to PO group (2.9 Gy vs 1.1 Gy, *P* = .054).

**Table 2.** Weight Changes at Weeks 3 and 6 in Bulky and Postoperative Groups.^a,b^

Parameter	Mean Percentage Change From Baseline (Range)						*P* Value for ifference Between B Versus PO	
	B			PO				
	Week 3	Week 6	*P* Value	Week 3	Week 6	*P* Value	Week 3	Week 6
Weight	−4.9% (4 to −10)	−8.6% (0 to −15)	<.001	−2.8% (2 to −8)	−4.7% (2 to −10)	.043	>.05	(.053)

Abbreviations: B, bulky disease; PO, postoperative.

^a^ *P* value from 2-way analysis of variance (ANOVA) model with 1 fixed factor (group), 1 repeated-measures factor (week), and their interaction (group × week).

^b^ *P* > .05: not significant. *P* ≤.05: significant difference within groups, week 3 versus 6, or between groups, B versus PO, at a specific week (3 or 6).

**Table 3.** Volumetric Changes at Weeks 3 and 6 in Bulky and Postoperative Groups.^a,b^

Parameter	Mean Percentage Change From Baseline (range)						*P* Value for Difference Between B Versus PO	
	B			PO				
	Week 3	Week 6	*P* Value	Week 3	Week 6	*P* Value	Week 3	Week 6
CTV-HR volume	−7.2% (3 to −18)	−12.8% (7 to −29)	.043	−7.8% (0 to −18)	−10.9% (−3 to −20)	> .05	> .05	> .05
CTV-LR volume	−4.1% (15 to −18)	−7.9% (3 to −17)	(.058)	−6.4% (0 to −14)	−6.4% (3 to −18)	> .05	> .05	> .05
RP volume	−19.0% (−4 to −32)	−30.9% (−20 to −52)	<.001	−16.6% (−5 to −44)	−25.3% (−3 to −41)	.005	> .05	> .05
LP volume	−18.2% (−3 to −28)	−30.1% (−11 to −43)	<.001	−10.0% (−5 to −17)	−23.1% (−7 to −36)	<.0001	.008	.04

Abbreviations: B, bulky disease; CTV-HR, high-risk clinical target volumes; CTV-LR, low-risk clinical target volumes; LP, left parotid; RP, right parotid; PO, postoperative.

^a^ *P* value from 2-way analysis of variance (ANOVA) model with 1 fixed factor (group), 1 repeated-measures factor (week), and their interaction (group × week).

^b^ *P* > .05: not significant. *P* ≤ .05: significant difference within groups, week 3 versus 6, or between groups, B versus PO, at a specific week (3 or 6).

**Table 4.** Dosimetric Changes at Weeks 3 and 6 in Bulky and Postoperative Groups.^a,b^

Parameter	Mean Percentage Change From Baseline (Range)						*P* Value for Difference Between B Versus PO	
	B			PO				
	Week 3	Week 6	*P* Value	Week 3	Week 6	*P* Value	Week 3	Week 6
CTV-HR mean dose	1.9% (6 to −1)	2.4% (6 to 0)	>.05	1.6% (3 to 0)	1.8% (4 to −17)	> .05	> .05	> .05
CTV-LR mean dose	1.2% (3 to −1)	2.4% (5 to −1)	.042	1.1% (3 to −18)	1.7% (6 to −25)	> .05	>.05	> .05
RP mean dose	15.2% (71 to −8)	16.4 % (76 to −30)	>.05	16.3% (89 to −13)	9.1% (70 to −25)	> .05	> .05	> .05
LP mean dose	16.1% (50 to −2)	15.8% (60 to −11)	>.05	6.3% (51 to −18)	10.4% (56 to −18)	> .05	> .05	>.05
SC maximum dose	0.08% (21 to 0)	0.06% (25 to −1)	>.05	0.03% (46 to −1)	0.04% (13 to −1)	> .05	(.052)	> .05

Abbreviations: B, bulky disease; CTV-HR, high-risk clinical target volumes; CTV-LR, low-risk clinical target volumes; LP, left parotid; RP, right parotid; PO, postoperative; SC, spinal cord.

^a^ *P* value from 2-way analysis of variance (ANOVA) model with 1 fixed factor (group), 1 repeated-measures factor (week), and their interaction (group × week).

^b^ *P* > .05: not significant. *P* ≤ .05: significant difference within groups, week 3 versus 6, or between groups, B versus PO, at a specific week (3 or 6).

**Table 5.** Type and Number of Dose Deviation at Weeks 3 and 6 in Bulky and Postoperative Groups.

Type of Event	PO (n = 11)	B (n = 11)	B Versus PO *P* Value^a^
	n (%)	n (%)	
Spinal cord maximum dose >45 Gy	1 (9.1)	2 (18.2)	1.000
Right parotid mean dose >26 Gy	--	3 (27.3)	.214
Left parotid mean dose >26 Gy	--	5 (45.5)	.035
CTV-HR coverage <95%	--	3 (27.3)	.214
CTV-LR coverage <95%	1 (9.1)	5 (45.5)	.149
CTV HR and/or LR coverage <95%	1 (9.1)	6 (54.1)	.063
At least 1 event	2 (18.2)	8 (72.7)	.030

Abbreviations: B, bulky disease; CTV-HR, high-risk clinical target volumes; CTV-LR, low-risk clinical target volumes; PO, postoperative.

^a^ *P* value from Fisher exact test.

### ART Need by Group

The proportion of patients with at least 1 dose deviation trigging ART was higher in group B than in group PO: 72.7% versus 18.2% (*P* = .03) overall. In group B, 8 of 11 patients had the following triggering events: overdose in SC (n = 2), RP (n = 3), LP (n = 5), or <95% coverage in CTV-HR (n = 3) or CTV-LR (n = 5). Such events occurred in 2 of 11 patients in the PO group: SC overdose (n = 1) and CTV-LR underdose (n = 1). Comparing group B versus PO at the specific weeks, the deviations were 54.5% versus 9.1% (*P* = .064) at week 3 and 63.6% versus 18.2% (*P* = .081) at week 6. Table 5 summarizes the type and number of deviations that otherwise would signal the need for ART. Although the CTV-HR and CTV-LR mean dose increase during treatment was not statistically different between both groups, the coverage of the target volume fell below 95% in 6 patients with B versus 1 PO patient (*P* = .064). In regard to OARs, the volume shrinkage and/or weight loss in group B resulted in surpassing the dose–volume constraints of the initial IMRT plan for parotid dose (8 B patients versus 0 PO patients, *P* = .001) and/or for SC dose (2 B vs 0 PO patients, *P* = .475).

### Predictive Factors of ART and Their Correlation

**Figure 1.**
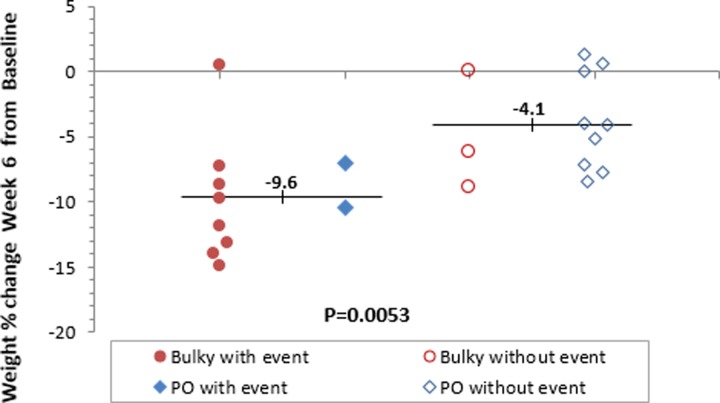
Significant weight loss among patients with at least 1 dose deviation triggering ART (solid circle or diamond) as compared with those without such event (open circle or diamond).

Pearson correlation analysis method was conducted to estimate linear correlation between clinical and dosimetric factors such as weight loss, parotids/target volume shrinkage, and dose distribution, considering both groups combined. The goal was to find impact of weight loss, for example, on dose distribution in an attempt to find an explanatory surrogate correlating with the need for ART. Percentage weight loss was strongly correlated with RP and LP volume shrinkage at both weeks 3 (Pearson correlation coefficient [*r*] = .56, *P* = .007, and *r* = 0.43, *P* = .043, respectively) and 6 (*r* = .52, *P* = .017, and *r* = .47, *P* = .028, respectively). As shown in Figure 1, a significant higher mean weight loss was found in patients who had a dose deviation triggering ART compared to the group of patient who did not have events, 9.6% versus 4.1% (*P* = .0053). Based on all 22 patients in both groups, at week 6, increase in SC maximum dose was strongly correlated with CTV-HR mean dose (*r* = .50, *P* = .018) and CTV-LR mean dose (*r* = .47, *P* = .028). Based on 10 patients with negative event (8 in B and 2 in PO), at week 6, increase in SC maximum dose was strongly correlated with CTV-HR mean dose (*r* = .62, *P* = .053) and CTV-LR mean (*r* = .48, *P* = .158), but given the small sample size, none reach significance at the 5% level.

## Discussion

To our knowledge, this is the first study that addresses dosimetric and volumetric changes in PO patients with HNC using 2 intratreatment CT image acquisitions in comparison with patients with B. In our study, the patients with B having HNC experienced significant changes with a magnitude similar to those described in other published series.^3,6,12^ While the PO group displayed significant volumetric changes in the parotid volumes, similar changes were not seen, as expected, in the CTV-HR and CTV-LR volumes. This confirms that the reduction in tumor burden during treatment often seen in patients with HNC having B partially explains the volume/dose dynamics and consequently the larger magnitude of benefit in this patient population as suggested by the study of Capelle *et al*.^10^ Due to the small sample size, the dosimetric alterations in group B were not statistically different from those in the PO group. However, focusing the analysis on the occurrence of dose deviations that would clinically mandate ART revealed that 72.7% of group B would benefit from an adaptive replan; a figure similar to 65% found in the study by Ahn *et al*.^12^ This is in contrast to only 18.2% dose deviations seen in the PO group, demonstrating a less frequent indication for ART in this patient population.

This study attempted to investigate the relative effects of weight loss and/or volumetric changes to explain the dosimetric difference between the B and PO groups. Ultimately, we aimed to detect clinically relevant parameters that would identify patients benefitting from ART in routine clinical practice. The mean percentage decrease in weight comparing week 3 and 6 to baseline was statistically significant in the B (4.9% and 8.6%, *P* ≤ .001) and PO groups (2.8% and 4.7%, *P* = .043). The magnitude of weight loss was almost twice as high in B compared to PO and, in combination with tumor shrinkage, could explain the greater number of events signaling an indication for ART in the B group. The patients who had events prompting ART had a mean weight loss of 9.6%, while those without this event had mean weight loss of 4.1% (*P* = .0053). Our study found a higher percentage weight loss was more likely to occur in the B group rather than the PO group.

Nevertheless, the correlation coefficient suggested a strong association between weight loss and parotid volume shrinkage in both groups analogous to the study by Barker *et al*.^2^ The additive effect of parotid volume shrinkage linked to weight loss and the decrease in the tumor volume might have led to dose deviations of larger magnitude seen more frequently in patients with B, rather than PO patients, and hence suggesting ART benefit in that group.^10^ In parallel to the study by Hansen *et al*,^3^ our data could not confirm weight loss, with its close correlation with volume changes,^12^ as the sole surrogate to initiate ART. Optimum image acquisition timing and/or frequency guidelines for replanning are still lacking. In this article, we attempted to find out the optimum timing to initiate ART. Bhide and colleagues prospectively followed 20 patients with HNC receiving concurrent ChRT using weekly CT. They demonstrated that week 2 is the time when dosimetric/volumetric changes are most significant.^13^ On the other hand, Wang and his colleagues illustrated that patients with nasopharynx cancer derive a significant benefit if replanning is done before fraction 25.^6^ Accordingly, we chose weeks 3 and 6 to detect changes at the most appropriate timing suggested by the literature while maintaining a compromise between single and weekly CT imaging. From a practical point of view, early replanning offers the ability to avoid plan degradation due to anatomical changes and the ability to limit cumulative dose deviation. Our data show that volumetric and dosimetric changes continue to occur during weeks 3 and 6. Hence, an optimal time point for initiating ART could not be recommended at week 3 versus week 6 based on our analysis. Although Wu *et al* showed that parotid sparing improves by 3% for 1 adaptive replanning during midcourse compared to a 6% improvement if replanning is performed 6 times during the treatment course,^7^ weekly replanning would be not feasible without the availability of image registration software to allow automation of the ART process. Overcoming these limitations with frequent adoption of onboard volumetric imaging such cone beam CT scans with heterogeneity correction, soft registration software, and process automation through specific dose deviation thresholds set to initiate the replanning algorithm offers a practical approach that must be weighed against excessive dose exposure and clinical therapeutic gain.

Our study included changes in CTV, rather than PTV, as an end point as it is a direct marker of tumor volume expansion and/or weight loss instead of setup error that would introduce other confounding elements.^14^ In addition, the prescription dose for CTV-HR in group B was 59.4 Gy, very similar to the 60 Gy CTV-HR prescription in the PO group to avoid having different dose parameters as a factor that would complicate the interpretation of results. Our data included some limitations, namely the manual repeat contours which may over- or underestimate the tissue changes due to inter- and intraobserver variability.^15^ Deformable registration software, although not free of limitation, might have been more consistent and clinically reliable.^16^ Another limitation of our study is the inherent error in image registration, which would accentuate the observed dosimetric differences.

The sample size potentially underpowered the significant difference between the comparison groups. Based on the trends found in our data, accruing a remarkably larger patient cohort was required to illustrate significant difference without necessarily altering the conclusion. The pilot nature of this study and the general trend in ART studies aiming at distinguishing optimum use of precise radiotherapy delivery dictated the appropriateness of the sample size.

Another limitation in our study that the actual cumulative dose delivered to the target and normal tissue volumes and its impact on the clinical outcome was not addressed. These 2 end points, not specified in our protocol, mandate daily imaging and long-term follow-up. The actual cumulative dose is a true reflection on the magnitude of dose deviation from the intended prescription and the actual benefit of ART.^5^ Nevertheless, the clinically driven dosimetric findings in our study provide an estimate of the amount of dose deviation without replanning policy and hint to a threshold for selecting patients benefiting the most from ART. Automation of the ART process by setting dose constraints detected by deformable registration software in rescanned imaging would allow early detection of dose deviation with early replanning, leading to a better cumulative dose or at least as close to our intended prescription as possible.^8^ With the wide availability of image guidance, implementation of practical ART policy may improve precision; however, whether the optimization and automation of cost, time, and labor-intensive ART process would result in meaningful clinical gain remain to be established.

## Conclusion

Serial change in volume and the resulting dosimetric changes in the target volumes and the OARs during head and neck chemo-IMRT occur both in patients with B and PO patients. However, the magnitude of these changes is more significant in patients with B having HNC, implying that an adaptive approach may have maximum benefit in this patient population. Significant volume and dose alteration occurs in a continuous fashion, negating the notion that ART should be initiated at a specific time point during the IMRT treatment process. Our data doesn’t support the routine use of ART in PO patients with HNC. However, significant weight loss exceeding 10% of the body weight can signal those patients at high risk of dosimetric changes, thus selecting them for optimum ART use. Further prospective studies are needed to confirm this finding.

## Supplementary Material

Supplementary material

## Abbreviations

B: bulky disease
ChRT: chemoradiotherapy
GTV: gross tumor volume
CTV-HR: high-risk clinical target volume
HNC: head and neck cancer
IMRT: intensity-modulated radiotherapy
CTV-LR: low-risk clinical target volume
LP: left parotid
PTV: planning target volume
PO: postoperative
PTV: planning target volume
PTV-HR: high-risk planning target volume
PTV-LR: low-risk planning target volume
RP: right parotid
SC: spinal cord.
