# Supplementary material for: Prospective Pilot Study Comparing the Need for Adaptive Radiotherapy in Unresected Bulky Disease and in Postoperative Patients With Head and Neck Cancer
Source: Technol Cancer Res Treat. 2017 Jul 3;16(6):1014–21. doi: 10.1177/1533034617717624 (PMC5762062; doi:10.1177/1533034617717624)
Supplement: Supplementary material [file Appendix_MAHMOUD.docx]

| Appendix Table 1: Weight changes at weeks 3 and 6 in Bulky and Post-operative groups | | | | | | |
| --- | --- | --- | --- | --- | --- | --- |
| Parameter | Mean Change from Baseline in pounds (range) | | | | | |
|  | B | | | PO | | |
|  | Week 3 | Week6 | P-value | Week 3 | Week 6 | P-value |
| Weight | -11 (7,-21) | -18(1,-36) | <.001 | -5(0,-12) | -9(1,-20) | 0.043 |
| P-value from two-way ANOVA model with one fixed factor (group), one repeated measures factor (week), and their interaction (group × week).  P>0.05: Not significant (P-value >0.05). P-value ≤0.05 is considered significant difference within-group, week 3 vs. 6, or between groups, B vs. PO, at a specific week (3 or 6). | | | | | | |

| Appendix Table 2: Volumetric changes at weeks 3 and 6 in Bulky and Post-operative groups | | | | | | |
| --- | --- | --- | --- | --- | --- | --- |
| Parameter | Mean Change from Baseline in cubic centimeter (range) | | | | | |
|  | B | | | PO | | |
|  | Week 3 | Week 6 | P-value | Week 3 | Week 6 | P-value |
| CTV-HR volume | -12 (4,-27) | -19 (21,-52) | 0.043 | -17 (0,-35) | -29 (-3,-121) | P>0.05 |
| CTV-LR volume | -8 (24,-42) | -15 (2,-42) | (0.058) | -8 (-1,-20) | -9(7,-29) | P>0.05 |
| RP volume | -5(-1,-12) | -8(-2,-17) | <.001 | -4(-1,-9) | -7(-1,-18) | 0.005 |
| LP volume | -4(-1,-10) | -7(-3,-16) | <.001 | -3(-1,-7) | -6(-1,-16) | <.0001 |
| *Abbreviations:* CTV-HR= CTV high risk; CTV-LR= CTV low risk; RP= right parotid; LP= left parotid.  P-value from two-way ANOVA model with one fixed factor (group), one repeated measures factor (week), and their interaction (group × week).  P>0.05: Not significant (P-value >0.05). P-value ≤0.05 is considered significant difference within-group, week 3 vs. 6, or between groups, B vs. PO, at a specific week (3 or 6). | | | | | | |

| Appendix Table 3: Dosimetric changes at weeks 3 and 6 in Bulky and Post-operative groups | | | | | | |
| --- | --- | --- | --- | --- | --- | --- |
| Parameter | Mean Change from Baseline in Gy (range) | | | | | |
|  | B | | | PO | | |
|  | Week 3 | Week 6 | P-value | Week 3 | Week 6 | P -value |
| CTV-HR mean dose | 1(4,-1) | 1(4,0) | P>0.05 | 1(2,0) | 0(3,-10) | P>0.05 |
| CTV-LR mean dose | 0(2,0) | 0(3,0) | 0.042 | 0(1,-11) | -1(3,-16) | P>0.05 |
| RP mean dose | 4(14,-2) | 3(15,-10) | P>0.05 | 3(11,-3) | 1(9,-6) | P>0.05 |
| LP mean dose | 3(9,-1) | 3(11,-3) | P>0.05 | 1(7,-4) | 1(8,-5) | P>0.05 |
| SC maximum dose | 4(9,0) | 3(8,-1) | P>0.05 | 2(15,0) | 2(5,-1) | P>0.05 |
| *Abbreviations:* CTV-HR= CTV high risk; CTV-LR= CTV low risk; RP= right parotid; LP= left parotid; SC= spinal cord.  P-value from two-way ANOVA model with one fixed factor (group), one repeated measures factor (week), and their interaction (group × week).  p>0.05: Not significant (P-value >0.05). P-value ≤0.05 is considered significant difference within-group, week 3 vs. 6, or between groups, B vs. PO, at a specific week (3 or 6). | | | | | | |

**Appendix Table 4. Comparisons of weight at weeks 1, 3, and 6**

| **Weight (lbs)** | **Bulky**  **(n=11)** | | | **Post-operative**  **(n=11)** | | | **P-value** |
| --- | --- | --- | --- | --- | --- | --- | --- |
|  | **Mean** | SD | SE | **Mean** | SD | SE |  |
| Week 1 | **194.7** | 36.6 | 11.0 | **166.7** | 39.9 | 12.0 |  |
| Week 3 | **184.6** | 31.8 | 9.6 | **162.6** | 41.5 | 12.5 |  |
| Week 6 | **177.2** | 28.9 | 8.7 | **159.1** | 39.5 | 11.9 |  |
| **% change from week 1** |  |  |  |  |  |  |  |
| Week 3 | **-4.9** | 4.3 | 1.3 | **-2.8** | 3.3 | 1.0 | P>0.05 |
| Week 6 | **-8.6** | 5.2 | 1.6 | **-4.7** | 3.9 | 1.2 | (0.053) |
| **P-value** | <.001 |  |  | 0.043 |  |  |  |
| P>0.05: Not significant (p>0.05) difference between corresponding % change means.  Note: All means of % change relative to week 1 (baseline) are significantly different from zero (p≤0.05). | | | | | | | |


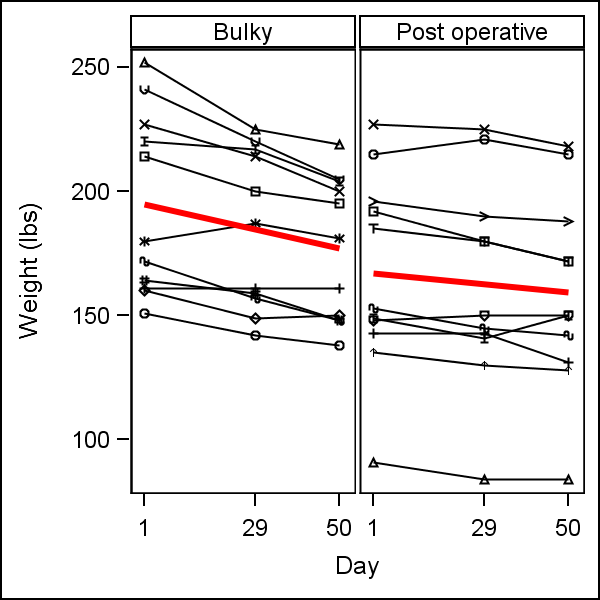

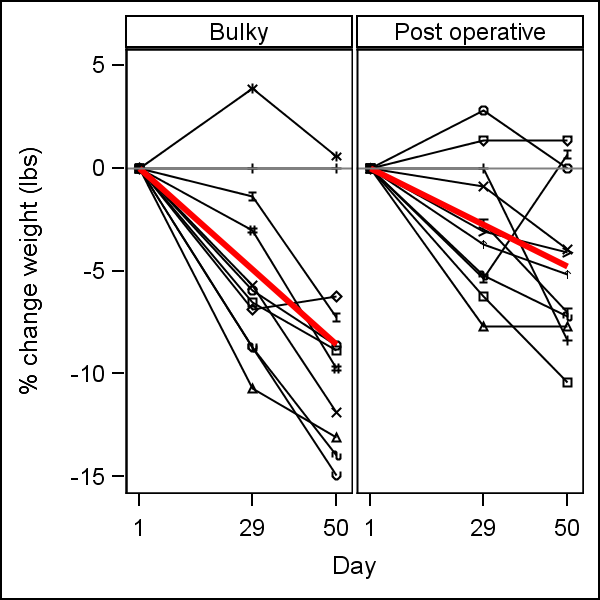


Black: individual patient data. Red: linear regression ignoring longitudinal nature of patient data.

**Appendix Table 5. Comparisons CTV high risk volume at weeks 1, 3, and 6**

| **CTV high risk volume** | **Bulky**  **(n=11)** | | | **Post-operative**  **(n=11)** | | | | **P-value** |
| --- | --- | --- | --- | --- | --- | --- | --- | --- |
|  | **Mean** | SD | SE | **Mean** | SD | | SE |  |
| Week 1 | **138.1** | 68.5 | 20.7 | **247.6** | 146.0 | | 44.0 |  |
| Week 3 | **127.3** | 62.2 | 18.8 | **228.1** | 135.6 | | 40.9 |  |
| Week 6 | **121.5** | 72.5 | 21.9 | **213.6** | 110.1 | | 33.2 |  |
| **% change from week 1** |  |  |  |  |  | |  |  |
| Week 3 | **-7.2** | 5.7 | 1.7 | **-7.8** | 5.3 | | 1.6 | P>0.05 |
| Week 6 | **-12.8** | 12.0 | 3.6 | **-10.9** | 6.9 | | 2.1 | P>0.05 |
| **P-value** | 0.043 |  |  | P>0.05 | |  |  |  |
| NS: Not significant (p>0.05) difference between corresponding % change means.  Note: All means of % change relative to week 1 (baseline) are significantly different from zero (p≤0.05). | | | | | | | | |


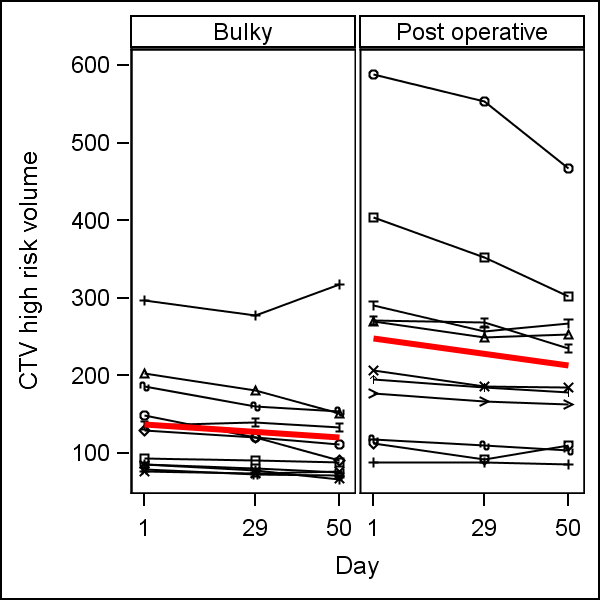

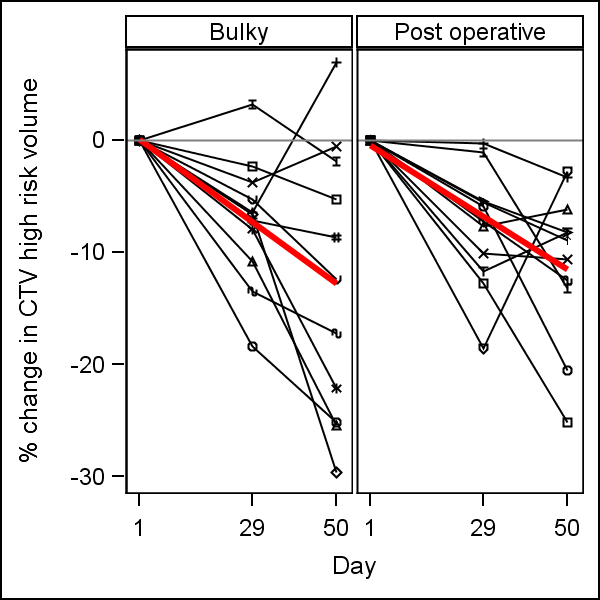


Black: individual patient data. Red: linear regression ignoring longitudinal nature of patient data.

**Appendix Table 6. Comparisons CTV low risk volume at weeks 1, 3, and 6**

| **CTV low risk volume** | **Bulky**  **(n=11)** | | | **Post-operative**  **(n=11)** | | | **P-value** |
| --- | --- | --- | --- | --- | --- | --- | --- |
|  | **Mean** | SD | SE | **Mean** | SD | SE |  |
| Week 1 | **166.3** | 86.4 | 26.0 | **148.3** | 88.9 | 26.8 |  |
| Week 3 | **158.0** | 78.0 | 23.5 | **139.7** | 87.9 | 26.5 |  |
| Week 6 | **150.0** | 75.6 | 22.8 | **139.4** | 84.7 | 25.5 |  |
| **% change from week 1** |  |  |  |  |  |  |  |
| Week 3 | **-4.1** | 10.2 | 3.1 | **-6.4** | 4.3 | 1.3 | P>0.05 |
| Week 6 | **-7.9** | 7.0 | 2.1 | **-6.4** | 5.7 | 1.7 | P>0.05 |
| **P-value** | (0.058) |  |  | P>0.05 |  |  |  |
| P>0.05: Not significant (p>0.05) difference between corresponding % change means.  Note: All means of % change relative to week 1 (baseline) are significantly different from zero (p≤0.05). | | | | | | | |


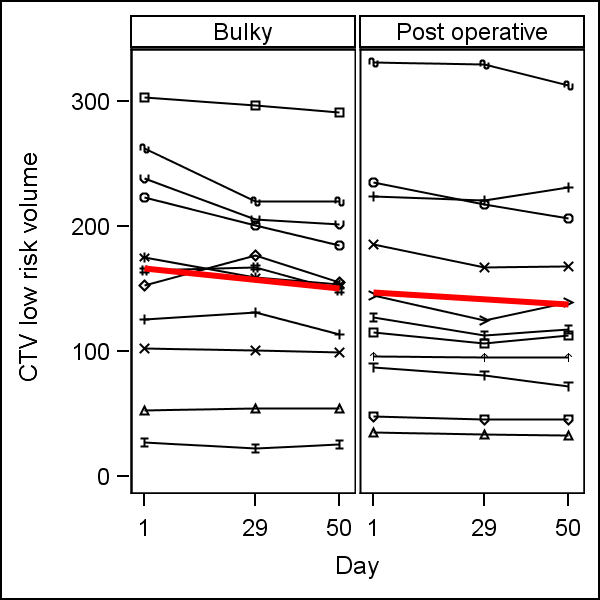

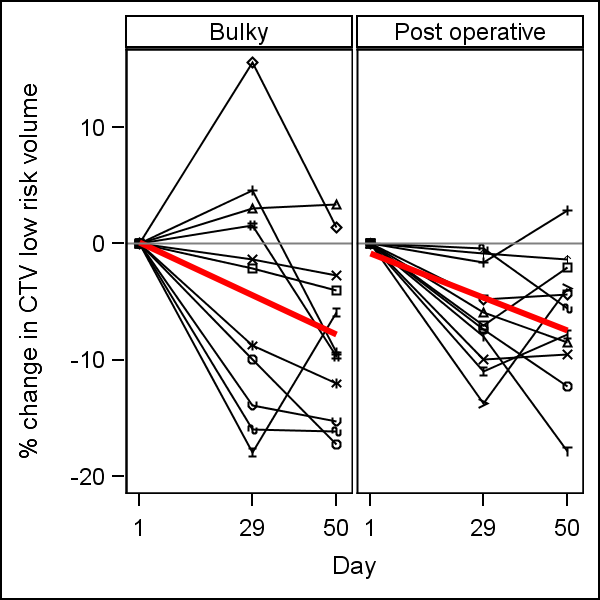


Black: individual patient data. Red: linear regression ignoring longitudinal nature of patient data.

**Appendix Table 7. Comparisons of right parotid volume at weeks 1, 3, and 6**

| **Right parotid volume** | **Bulky**  **(n=11)** | | | **Post-operative**  **(n=11)** | | | **P-value** |
| --- | --- | --- | --- | --- | --- | --- | --- |
|  | **Mean** | SD | SE | **Mean** | SD | SE |  |
| Week 1 | **25.8** | 10.4 | 3.1 | **26.4** | 7.2 | 2.3 |  |
| Week 3 | **20.6** | 8.4 | 2.5 | **22.1** | 6.7 | 2.1 |  |
| Week 6 | **17.7** | 7.8 | 2.4 | **19.5** | 4.5 | 1.4 |  |
| **% change from week 1** |  |  |  |  |  |  |  |
| Week 3 | **-19.0** | 10.5 | 3.2 | **-16.6** | 12.0 | 3.8 | P>0.05 |
| Week 6 | **-30.9** | 12.0 | 3.6 | **-25.3** | 10.2 | 3.2 | P>0.05 |
| **P-value** | <.001 |  |  | 0.005 |  |  |  |
| P>0.05: Not significant (p>0.05) difference between corresponding % change means.  Note: All % change means significantly different from zero (p≤0.05), therefore significant change at weeks 3 and 6 relative to baseline (week1). | | | | | | | |


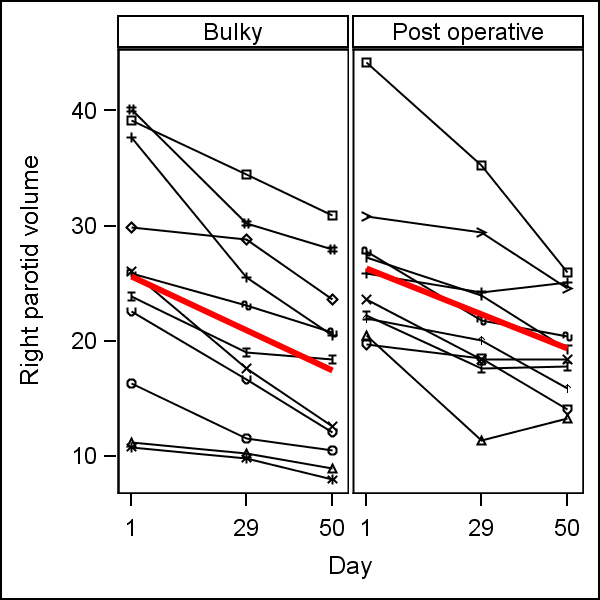

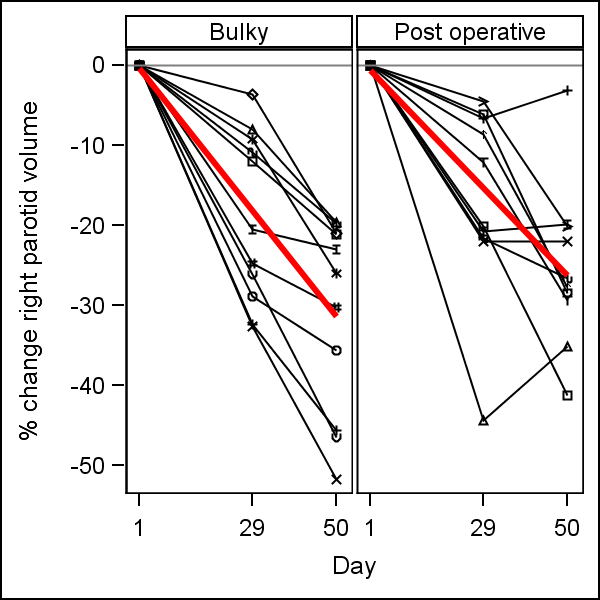


Black: individual patient data. Red: linear regression ignoring longitudinal nature of patient data.

**Appendix Table 8. Comparisons of left parotid volume at weeks 1, 3, and 6**

| **Left parotid volume** | **Bulky**  **(n=11)** | | | **Post-operative**  **(n=11)** | | | **P-value** |
| --- | --- | --- | --- | --- | --- | --- | --- |
|  | **Mean** | SD | SE | **Mean** | SD | SE |  |
| Week 1 | **24.3** | 9.0 | 2.7 | **24.0** | 9.0 | 2.7 |  |
| Week 3 | **19.8** | 7.7 | 2.3 | **21.4** | 7.4 | 2.2 |  |
| Week 6 | **17.0** | 7.0 | 2.1 | **18.1** | 5.6 | 1.7 |  |
| **% change from week 1** |  |  |  |  |  |  |  |
| Week 3 | **-18.2** | 8.7 | 2.6 | **-10.0** | 4.2 | 1.3 | 0.008 |
| Week 6 | **-30.1** | 8.8 | 2.7 | **-23.1** | 6.7 | 2.0 | 0.040 |
| **P-value** | <.0001 |  |  | <.0001 |  |  |  |
| P>0.05: Not significant (p>0.05) difference between corresponding % change means.  Note: All means of % change relative to week 1 (baseline) are significantly different from zero (p≤0.05). | | | | | | | |


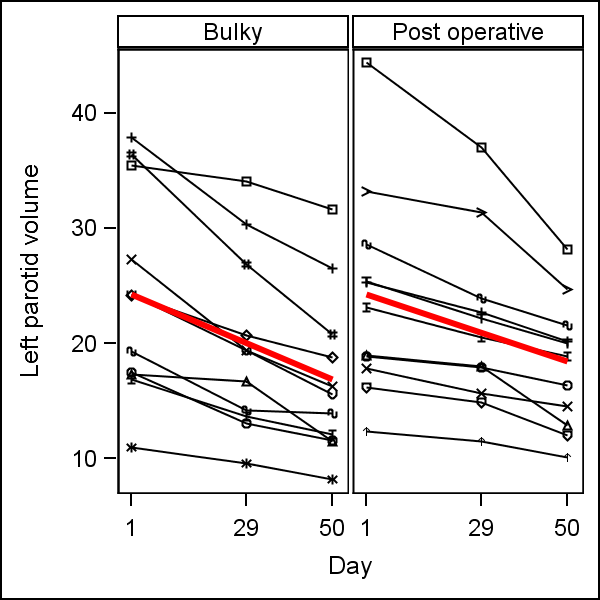

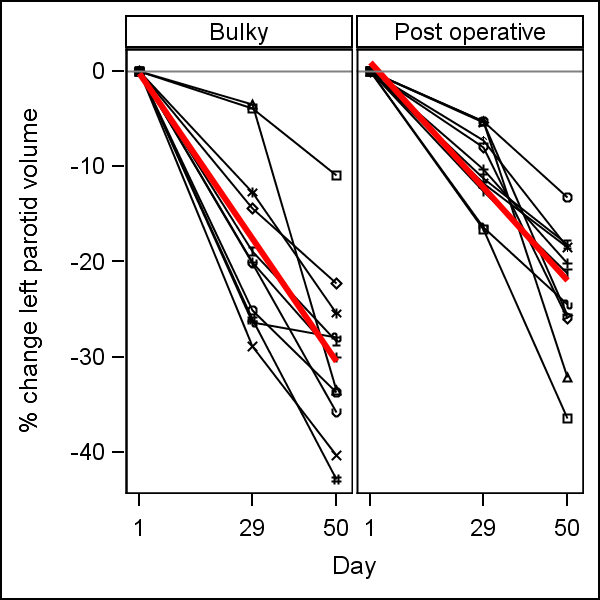


Black: individual patient data. Red: linear regression ignoring longitudinal nature of patient data.

**Appendix Table 9. Comparisons of CTV high risk mean dose at weeks 1, 3, and 6**

| **CTV high risk mean dose** | **Bulky**  **(n=11)** | | | **Post-operative**  **(n=11)** | | | **P-value** |
| --- | --- | --- | --- | --- | --- | --- | --- |
|  | **Mean** | SD | SE | **Mean** | SD | SE |  |
| Week 1 | **65.7** | 4.3 | 1.3 | **64.6** | 3.9 | 1.2 |  |
| Week 3 | **67.0** | 3.8 | 1.2 | **65.7** | 4.3 | 1.3 |  |
| Week 6 | **67.3** | 4.3 | 1.3 | **65.8** | 4.5 | 1.4 |  |
| **% change from week 1** |  |  |  |  |  |  |  |
| Week 3 | **1.9** | 1.8 | 0.5 | **1.6** | 0.8 | 0.2 | P>0.05 |
| Week 6 | **2.4** | 2.0 | 0.6 | **1.8** | 1.2 | 0.4 | P>0.05 |
| **P-value** | P>0.05 |  |  | P>0.05 |  |  |  |
| P>0.05: Not significant (p>0.05) difference between corresponding % change means.  Note: All means of % change relative to week 1 (baseline) are significantly different from zero (p≤0.05). | | | | | | | |


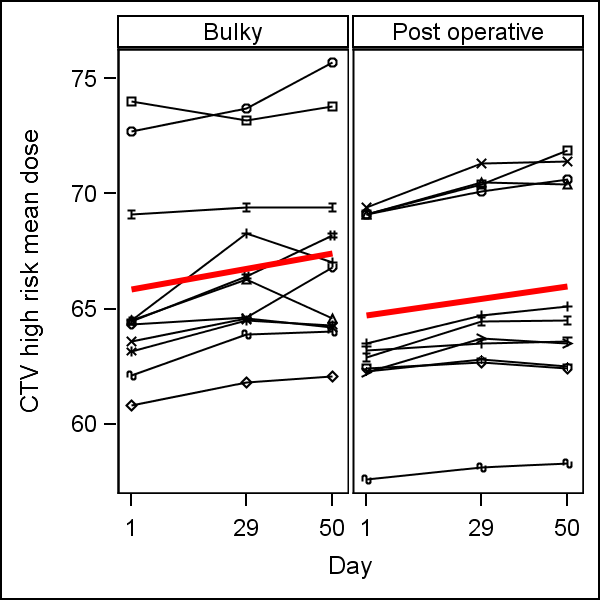

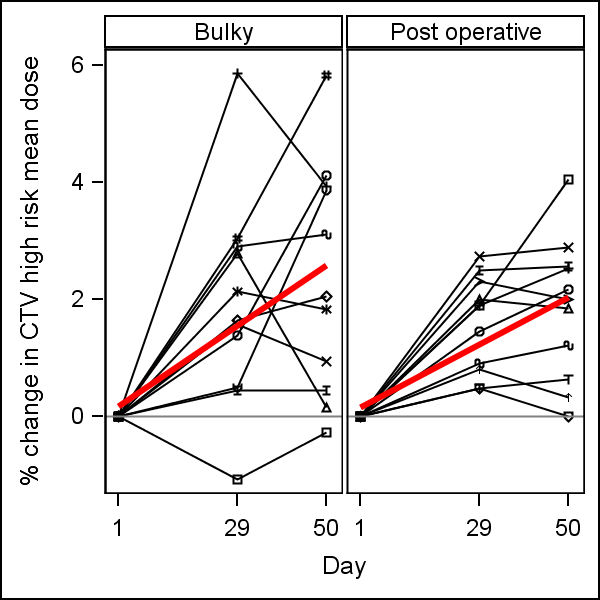


Black: individual patient data. Red: linear regression ignoring longitudinal nature of patient data.

**Appendix Table 10. Comparisons of CTV low risk mean dose at weeks 1, 3, and 6**

| **CTV low risk mean dose** | **Bulky**  **(n=11)** | | | **Post-operative**  **(n=11)** | | | **P-value** |
| --- | --- | --- | --- | --- | --- | --- | --- |
|  | **Mean** | SD | SE | **Mean** | SD | SE |  |
| Week 1 | **56.4** | 4.2 | 1.3 | **56.5** | 1.9 | 0.6 |  |
| Week 3 | **57.1** | 4.6 | 1.4 | **57.1** | 2.1 | 0.6 |  |
| Week 6 | **57.8** | 4.9 | 1.5 | **57.5** | 2.2 | 0.7 |  |
| **% change from week 1** |  |  |  |  |  |  |  |
| Week 3 | **1.2** | 1.2 | 0.4 | **1.1** | 0.9 | 0.3 | P>0.05 |
| Week 6 | **2.4** | 2.0 | 0.6 | **1.7** | 1.9 | 0.6 | P>0.05 |
| **P-value** | 0.042 |  |  | P>0.05 |  |  |  |
| P>0.05: Not significant (p>0.05) difference between corresponding % change means.  Note: All means of % change relative to week 1 (baseline) are significantly different from zero (p≤0.05). | | | | | | | |


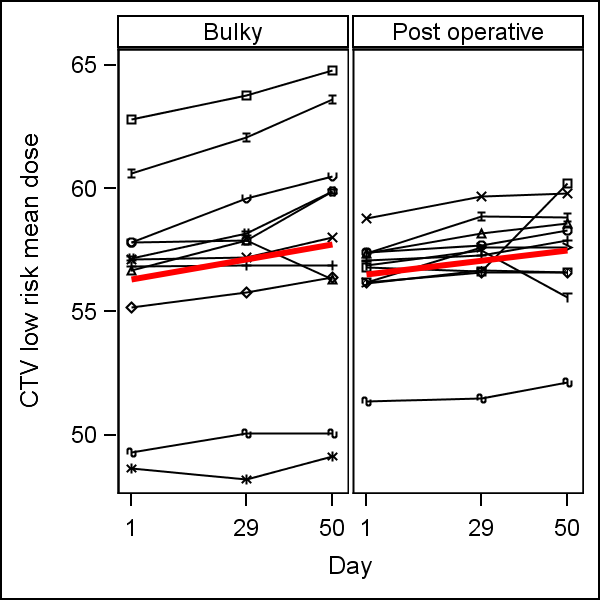

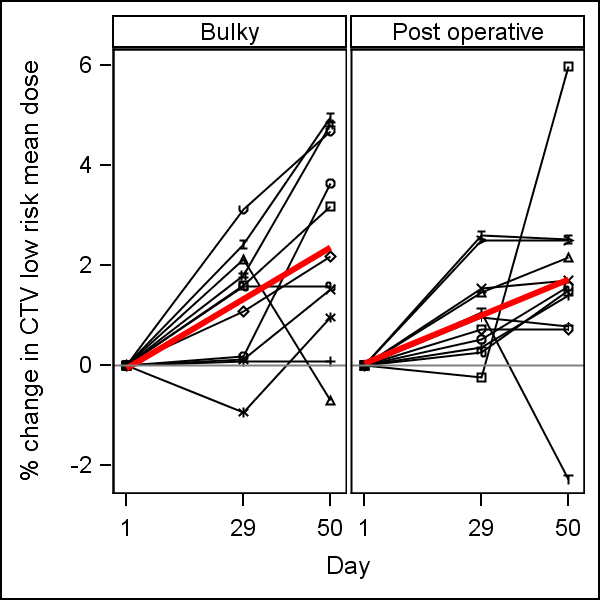


Black: individual patient data. Red: linear regression ignoring longitudinal nature of patient data.

**Appendix Table 11. Comparisons of right parotid mean dose at weeks 1, 3, and 6**

| **Right parotid mean dose** | **Bulky**  **(n=11)** | | | **Post-operative**  **(n=11)** | | | **P-value** |
| --- | --- | --- | --- | --- | --- | --- | --- |
|  | **Mean** | SD | SE | **Mean** | SD | SE |  |
| Week 1 | **25.2** | 7.2 | 2.2 | **26.4** | 14.4 | 4.6 |  |
| Week 3 | **28.5** | 7.1 | 2.1 | **28.9** | 13.8 | 4.4 |  |
| Week 6 | **28.4** | 7.4 | 2.2 | **27.7** | 15.0 | 4.7 |  |
| **% change from week 1** |  |  |  |  |  |  |  |
| Week 3 | **15.2^#^** | 22.9 | 6.9 | **16.3^#^** | 33.5 | 10.6 | P>0.05 |
| Week 6 | **16.4^#^** | 30.6 | 9.2 | **9.1^#^** | 28.0 | 8.9 | P>0.05 |
| **P-value** | P>0.05 |  |  | P>0.05 |  |  |  |
| P>0.05: Not significant (p>0.05) difference between corresponding % change means.  ^#^ The particular means of % change are not significantly different from zero (p>0.05). Thus, there are not significant changes at weeks 3 and 6 relative to week 1 (baseline). | | | | | | | |


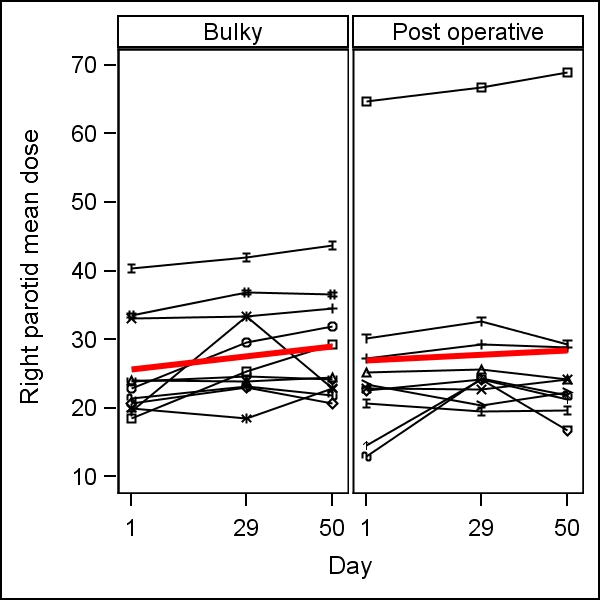

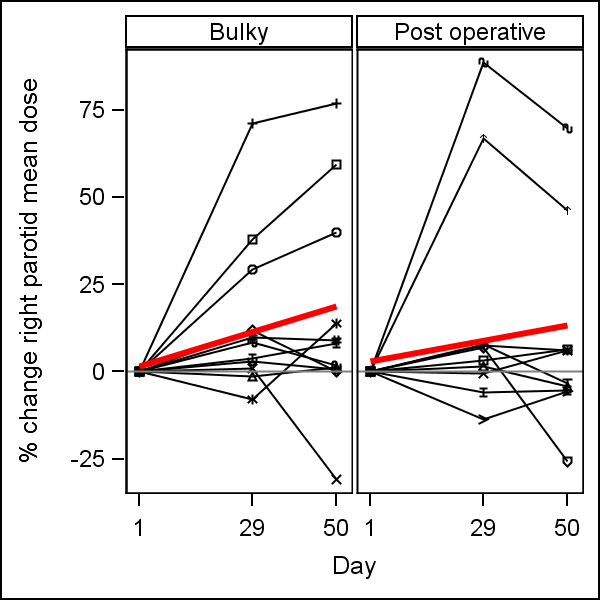


Black: individual patient data. Red: linear regression ignoring longitudinal nature of patient data.

**Table 12. Comparisons of left parotid mean dose at weeks 1, 3, and 6**

| **Left parotid mean dose** | **Bulky**  **(n=11)** | | | **Post-operative**  **(n=11)** | | | **P-value** |
| --- | --- | --- | --- | --- | --- | --- | --- |
|  | **Mean** | SD | SE | **Mean** | SD | SE |  |
| Week 1 | **25.2** | 8.1 | 2.5 | **21.5** | 4.6 | 1.4 |  |
| Week 3 | **28.7** | 7.7 | 2.3 | **22.2** | 3.5 | 1.1 |  |
| Week 6 | **28.5** | 7.8 | 2.4 | **23.0** | 2.9 | 0.9 |  |
| **% change from week 1** |  |  |  |  |  |  |  |
| Week 3 | **16.1** | 17.2 | 5.2 | **6.3^#^** | 21.0 | 6.3 | P>0.05 |
| Week 6 | **15.8** | 22.6 | 6.8 | **10.4^#^** | 20.8 | 6.3 | P>0.05 |
| **P-value** | P>0.05 |  |  | P>0.05 |  |  |  |
| P>0.05: Not significant (p>0.05) difference between corresponding % change means.  ^#^ The particular two means of % change are not significantly different from zero (p>0.05). The other two % change means are significantly different from zero (p≤0.05). | | | | | | | |


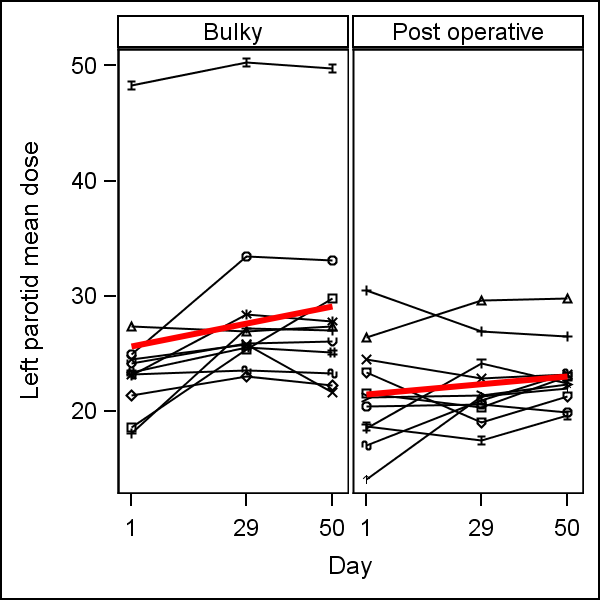

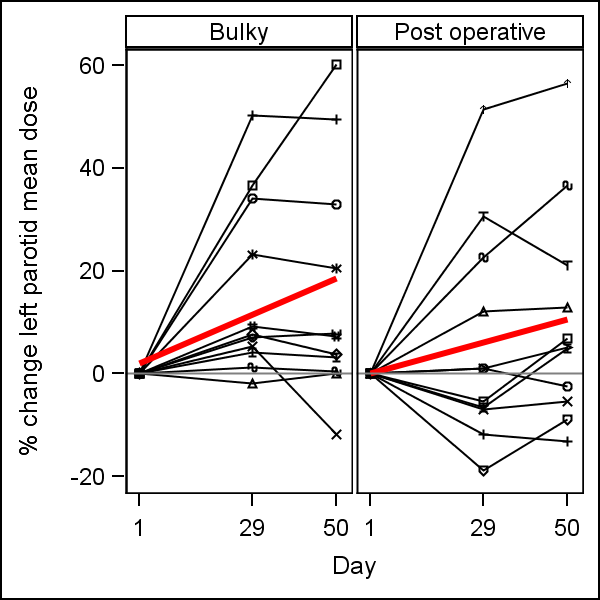


Black: individual patient data. Red: linear regression ignoring longitudinal nature of patient data.

**Table 13. Comparisons of spinal cord maximum dose at weeks 1, 3, and 6**

| **Spinal cord  maximum dose** | **Bulky**  **(n=11)** | | | **Post-operative**  **(n=11)** | | | | **P-value** |
| --- | --- | --- | --- | --- | --- | --- | --- | --- |
|  | **Mean** | SD | SE | **Mean** | SD | | SE |  |
| Week 1 | 39.0 | 4.0 | 1.2 | **40.7** | 2.9 | | 0.9 |  |
| Week 3 | 41.9 | 4.8 | 1.5 | **41.8** | 2.8 | | 0.9 |  |
| Week 6 | 41.4 | 4.6 | 1.4 | **42.4** | 2.8 | | 0.9 |  |
| **% change from week 1** |  |  |  |  |  | |  |  |
| Week 3 | 0.08 | 0.08 | 0.02 | **0.03^#^** | 0.03 | | 0.01 | (0.052) |
| Week 6 | 0.06 | 0.06 | 0.02 | **0.04** | 0.05 | | 0.02 | P>0.05 |
| **P-value** | P>0.05 |  |  | P>0.05 | |  |  |  |
| P>0.05: Not significant (p>0.05) difference between corresponding % change means.  ^#^ The particular mean of % change at week 4 is not significantly different from zero (p>0.05). The other three % change means are significantly different from zero (p≤0.05). | | | | | | | | |


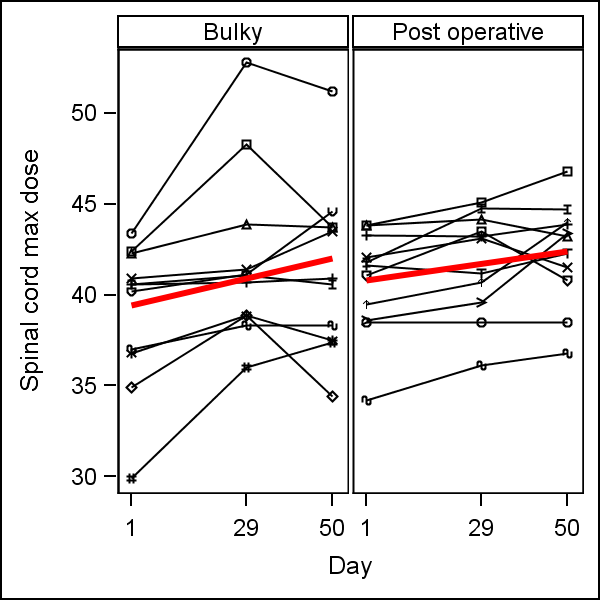

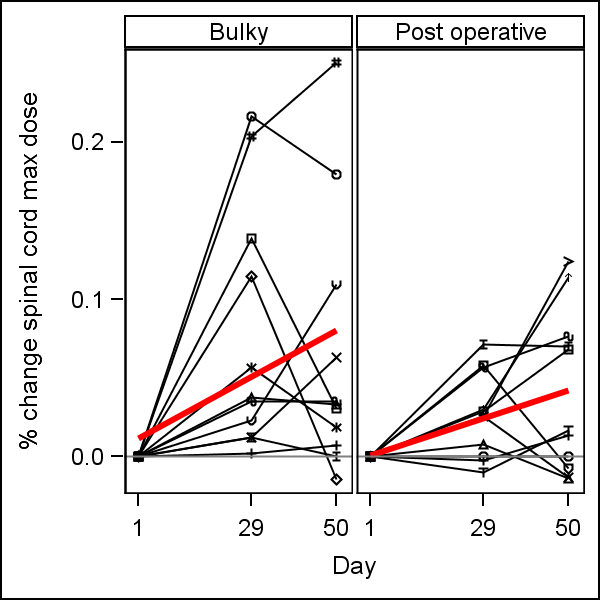


Black: individual patient data. Red: linear regression ignoring longitudinal nature of patient data.

------

**Statistical analysis:**

Volumetric and dosimetric features were obtained at weeks 1 (baseline), 3 and 6. Raw data and percentage change relative to baseline (= 100×change/baseline) were summarized as means, standard deviation and standard error. Percentage change variables at weeks 3 and 6 relative to baseline were analyzed under a two-way repeated measures ANOVA model using SAS Mixed procedure. Group (bulky or post operative) was considered a fixed factor and time (week 3 or 6) was treated as a repeated measures factor. Tests for comparison of groups at weeks 3 and 6, as well as within-group comparisons of % change for week 3 versus 6, were performed using contrasts. To evaluate the changes relative to baseline, we tested if each % change mean was significantly different from zero. The Fisher’s exact test was used for group comparison with respect to categorical outcomes.
